# Molecular Profiling of Breast Cancer Cell Lines Defines Relevant Tumor Models and Provides a Resource for Cancer Gene Discovery

**DOI:** 10.1371/journal.pone.0006146

**Published:** 2009-07-03

**Authors:** Jessica Kao, Keyan Salari, Melanie Bocanegra, Yoon-La Choi, Luc Girard, Jeet Gandhi, Kevin A. Kwei, Tina Hernandez-Boussard, Pei Wang, Adi F. Gazdar, John D. Minna, Jonathan R. Pollack

**Affiliations:** 1 Department of Pathology, Stanford University, Stanford, California, United States of America; 2 Department of Genetics, Stanford University, Stanford, California, United States of America; 3 Department of Pathology, Samsung Medical Center, Sungkyunkwan University School of Medicine, Seoul, South Korea; 4 Hamon Center for Therapeutic Oncology Research, University of Texas Southwestern Medical Center, Dallas, Texas, United States of America; 5 Division of Public Health Sciences, Fred Hutchinson Cancer Research Center, Seattle, Washington, United States of America; Roswell Park Cancer Institute, United States of America

## Abstract

**Background:**

Breast cancer cell lines have been used widely to investigate breast cancer pathobiology and new therapies. Breast cancer is a molecularly heterogeneous disease, and it is important to understand how well and which cell lines best model that diversity. In particular, microarray studies have identified molecular subtypes–luminal A, luminal B, ERBB2-associated, basal-like and normal-like–with characteristic gene-expression patterns and underlying DNA copy number alterations (CNAs). Here, we studied a collection of breast cancer cell lines to catalog molecular profiles and to assess their relation to breast cancer subtypes.

**Methods:**

Whole-genome DNA microarrays were used to profile gene expression and CNAs in a collection of 52 widely-used breast cancer cell lines, and comparisons were made to existing profiles of primary breast tumors. Hierarchical clustering was used to identify gene-expression subtypes, and Gene Set Enrichment Analysis (GSEA) to discover biological features of those subtypes. Genomic and transcriptional profiles were integrated to discover within high-amplitude CNAs candidate cancer genes with coordinately altered gene copy number and expression.

**Findings:**

Transcriptional profiling of breast cancer cell lines identified one luminal and two basal-like (A and B) subtypes. Luminal lines displayed an estrogen receptor (ER) signature and resembled luminal-A/B tumors, basal-A lines were associated with ETS-pathway and BRCA1 signatures and resembled basal-like tumors, and basal-B lines displayed mesenchymal and stem/progenitor-cell characteristics. Compared to tumors, cell lines exhibited similar patterns of CNA, but an overall higher complexity of CNA (genetically simple luminal-A tumors were not represented), and only partial conservation of subtype-specific CNAs. We identified 80 high-level DNA amplifications and 13 multi-copy deletions, and the resident genes with concomitantly altered gene-expression, highlighting known and novel candidate breast cancer genes.

**Conclusions:**

Overall, breast cancer cell lines were genetically more complex than tumors, but retained expression patterns with relevance to the luminal-basal subtype distinction. The compendium of molecular profiles defines cell lines suitable for investigations of subtype-specific pathobiology, cancer stem cell biology, biomarkers and therapies, and provides a resource for discovery of new breast cancer genes.

## Introduction

Breast cancer, a leading cause of cancer death in women, is recognized to be a molecularly heterogeneous disease. Markers such as estrogen receptor (ER), progesterone receptor (PR) and ERBB2/HER2 are used for prognostication, and to stratify patients for appropriately targeted therapies [1].

More recently, DNA microarray studies have suggested a refined classification of breast cancer, distinguishing five major subtypes based on different patterns of gene expression, underlying DNA copy number alterations (CNAs), and associated clinical outcomes [2]–[5]. Luminal subtypes A and B are ER positive and share expression markers with the luminal epithelial layer of cells lining normal breast ducts. Luminal-A tumors are genetically simple (1q/16p gain) and are associated with favorable outcome, while luminal-B tumors exhibit high proliferation rates, frequent DNA amplification (e.g. 8q24/*MYC*), and less favorable prognosis. Basal-like tumors share expression markers with the underlying basal (myoepithelial) layer of normal breast ducts, are ER negative, exhibit frequent chromosome segmental gains/losses, and are associated with poor outcome in most studies. The ERBB2 subtype is associated with expression of genes co-amplified with *ERBB2* (encoding HER2) on chromosome cytoband 17q12, and the normal-like subtype shares expression patterns with normal breast tissue.

Breast cancer cell lines have been used widely to investigate breast cancer pathobiology, and to screen and characterize new therapeutics [6], [7]. Advantages of cell lines include the relative ease of pharmacologic and genetic manipulation, the variety of available functional assays, and, for some studies, the purity of the cancerous epithelial population (and absence of stromal cell contamination). However, while some investigators choose particular cell lines based on the known ER or HER2 status, many others rely on standard “workhorses” like MCF7 without regard to the particular tumor subtypes being modeled. The recent recognition of microarray molecular subtypes points to the need for additional consideration in cell line selection.

The goal of our study was to profile gene expression and CNAs genome-wide in a collection of 52 publicly-available and commonly-used breast cancer cell lines, in order to assess the relation of these cell lines to the recognized molecular subtypes of breast cancer, and to discover new candidate breast cancer genes and pathways.

## Materials and Methods

### Breast Cancer Cell Lines

184A1, BT20, BT474, BT483, BT549, Hs578T, hTERT-HME1, MCF7, MCF10A, MDA-MB134, MDA-MB157, MDA-MB175, MDA-MB231, MDA-MB361, MDA-MB436, MDA-MB453, MDA-MB468, SKBR3, T47D, UACC812, UACC893, ZR75-1 and ZR75-30 were obtained from ATCC (Manassas, VA, USA). EFM19 and EFM192A were obtained from DSMZ (Braunschweig, Germany). HCC38, HCC70, HCC202, HCC712, HCC1007, HCC1143, HCC1395, HCC1419, HCC1428, HCC1500, HCC1569, HCC1599, HCC1806, HCC1937, HCC1954, HCC2157, HCC2185, HCC2218, HCC2688 and HCC3153 were obtained from the cell repository of the Hamon Center for Therapeutic Oncology Research, UT Southwestern Medical Center (many are now available from ATCC). CAL51 was a kind gift from J. Gioanni from the Centre Antoine-Lacassagne, Nice, France. SUM44PE, SUM52PE, SUM102PT, SUM149PT and SUM190PT were kind gifts from Dr. Stephen P. Ethier (now available from Asterand, Detroit, MI). MCF10A was grown in MEGM media (Cambrex, East Rutherford, NJ). SUM52PE and SUM149PT were grown in Ham's F12 media with 5% FBS, supplemented with 5 µg/ml insulin and 1 µg/ml hydrocortisone. SUM44PE, SUM102PT and SUM190PT were grown in Ham's F12 with 0.1% BSA, supplemented with 5 µg/ml insulin, 1 µg/ml of hydrocortisone, 5 mM ethanolamine, 10 mM HEPES, 5 µg/ml transferrin, 10 nM of Triiodo Thyronin (T3) and 50 nM sodium selenite (10 ng/ml EGF was also included for SUM102PT). All other cell lines were grown in RPMI-1640 with 10% FBS and 1% Pen/Strep. Clinicopathological characteristics of cell lines are summarized in Table 1. A subset of cell lines (focused on the HCC series) was subjected to a more detailed molecular pathological characterization of *ESR1*, *PGR*, *ERBB2*, *EGFR* and *BRCA1*, as summarized in Table 2.

**Table 1:** Clinicopathological features of breast cancer cell lines.

Cell line	Subtype#	ER*	PR*	ERBB2/HER2*	Source€	Tumor type€
184A1	B	−	NA	−	RM	NA
BT20	A	−	−	−	PT	AC
BT474	L	+	+	+	PT	IDC
BT483	L	+	+	−	PT	IDC
BT549	B	−	−	−	PT	IDC
CAL51	B	−	NA	−	PE	AC
EFM19	L	+	+	−	PE	IDC
EFM192A	L	+	+	+	PE	AC
HCC38	B	−	−	−	PT	DC
HCC70	A	−	−	−	PT	DC
HCC202	L	−	−	+	PT	DC
HCC712	L	+	−	−	PT	DC
HCC1007	L	+	−	+	PT	DC
HCC1143	A	−	−	−	PT	DC
HCC1187	A	−	−	−	PT	DC
HCC1395	B	−	−	−	PT	DC
HCC1419	L	−	−	+	PT	DC
HCC1428	L	+	+	−	PE	Met AC
HCC1500	L	+	+	−	PT	DC
HCC1569	A	−	−	+	PT	Met C
HCC1599	A	−	−	−	PT	DC
HCC1806	NA	−	−	−	PT	Sq C
HCC1937	A	−	−	−	PT	DC
HCC1954	A	−	−	+	PT	DC
HCC2157	A	−	−	−	PT	NA
HCC2185	L	−	−	−	PE	Met LC
HCC2218	L	−	−	+	PT	DC
HCC2688	L	−	NA	−	PT	DC
HCC3153	A	−	−	−	PT	DC
HS578T	B	−	−	−	PT	C Sar
hTERT-HME1	B	−	NA	−	RM	NA
MCF7	L	+	+	−	PE	Met AC
MCF10A	B	−	−	−	RM	F
MDA134	L	+	−	−	PE	IDC
MDA157	B	−	−	−	PE	Med C
MDA175	L	+	−	−	PE	IDC
MDA231	B	−	−	−	PE	Met AC
MDA361	L	+	+	+	BR	Met AC
MDA436	B	−	−	−	PE	AC
MDA453	L	−	−	+¶	PE	Met C
MDA468	A	−	−	−	PE	Met AC
SKBR3	L	−	−	+	PE	AC
SUM44	NA	+	+	+	PE	ILC
SUM52	L	+	−	+	PE	Met C
SUM102	B	−	−	−	PE	IDC, apocrine
SUM149	B	−	−	−	PE	Inf
SUM190	L	−	−	+	PT	Inf
T47D	L	+	+	−	PE	IDC
UACC812	L	+	−	+	PT	IDC
UACC893	L	−	−	+	PT	IDC
ZR75-1	L	+	−	−	AF	IDC
ZR75-30	L	+	−	+	AF	IDC

Abbreviations: A = Basal A subtype; AC = adenocarcinoma; AF = ascites fluid; B = Basal B subtype; BR = brain; C Sar = carcinoma sarcoma; DC = ductal carcinoma; F = fibrocystic disease; IDC = invasive ductal carcinoma; Inf = inflammatory carcinoma; ILC = invasive lobular carcinoma; L = Luminal subtype; Med C = medullary carcinoma, Met AC = metastatic adenocarcinoma; Met C = metastatic carcinoma, Met LC = metastatic lobular carcinoma; NA = not available; PE = pleural effusion; PT = primary tumor; RM = reduction mammoplasty; Sq C = Squamous Carcinoma.

# Determined from this study.

* Determined from the ATCC (http://www.atcc.org) and DSMZ (http://www.dsmz.de) websites, and references therein, or from this study.

€ Determined from the ATCC and DSMZ websites, and references therein.

¶ *ERBB2* amplified but not highly expressed.

**Table 2:** Molecular pathological analysis of breast cancer cell line subset.

Cell line	Phenotype	BRCA1	Q-PCR# ERBB2	Q-RT-PCR*				IHC			Western			
				ESR1	PGR	ERBB2	EGFR	ESR1	PGR	ERBB2	ESR1	PGR	ERBB2	EGFR
HCC38	Triple neg		1.18	−	−	−	−	**−**	**−**	**−**	**−**	**−**	**−**	**−**
HCC70	Triple neg		0.37	−	−	−	+	**−**	**−**	**−**	**−**	**−**	**−**	**+**
HCC202	ERBB2 amp		**28.88**	**−**	**−**	**+**	**+**	**−**	**−**	**+**	**−**	**−**	**+**	**+**
HCC712	Hormone+		0.95	**+**	**−**	**−**	**−**	**+**	**+**		**+**	**−**	**−**	**−**
HCC1143	Triple neg		1.08	**−**	**−**	**−**	**+**	**−**	**−**	**−**	**−**	**−**	**−**	**+**
HCC1187	Triple neg		0.42	**−**	**−**	**−**	**−**		**−**	**−**	**−**	**−**	**−**	**+**
HCC1395	Triple neg		0.36	**−**	**−**	**−**	**−**	**−**	**−**	**−**	**−**	**−**	**−**	**−**
HCC1419	ERBB2 amp		**8.39**	**−**	**−**	**+**	**−**	**−**		**+**	**−**	**−**	**+**	**−**
HCC1428	Hormone+		0.20	**+**	**+**	**−**	**−**	**+**	**+**	**−**	**+**	**+**	**−**	**−**
HCC1500	Hormone+		0.38	**+**	**+**	**−**	**−**	**+**	**+**	**−**	**+**	**−**	**−**	**−**
HCC1569	ERBB2 amp		**33.75**	**−**	**−**	**+**	**+**	**−**	**−**	**+**	**−**	**−**	**+**	**+**
HCC1806	Triple neg		0.08	**−**	**−**	**−**	**+**	**−**	**−**	**−**	**−**	**−**	**−**	**+**
HCC1937	Triple neg	INS C 5382	0.33	**−**	**−**	**−**	**+**	**−**	**−**	**−**	**−**	**−**	**−**	**+**
HCC1954	ERBB2 amp		**45.01**	**−**	**−**	**+**	**+**	**−**	**−**	**+**	**−**	**−**	**+**	**+**
HCC2185	Triple neg		0.63	**−**	**−**	**−**	**+**	**−**	**−**		**−**	**−**	**−**	**+**
HCC3153	Triple neg	943 ins 10	0.64	**−**	**−**	**−**	**+**	**−**	**−**	**−**	**−**	**−**	**−**	**+**
MCF7	Hormone+		0.56	**+**	**−**	**−**	**−**				**+**	**−**	**−**	**−**
BT483	Hormone+		0.19	**+**	**+**	**−**	**−**				**+**	**+**	**−**	**−**
BT549	Triple neg		0.63	**−**	**−**	**−**	**+**				**−**	**−**	**−**	**+**
MDA157	Triple neg		0.76	**−**	**−**	**−**	**+**				**−**	**−**	**−**	**−**
MDA231	Triple neg		0.90	**−**	**−**	**−**	**+**				**−**	**−**	**−**	**+**
MDA453	Triple neg		3.88	**−**	**−**	**+**	**−**				**−**	**−**	**−**	**−**
MDA134	Hormone+		0.76	**+**	**−**	**−**	**−**				**+**	**−**	**−**	**−**
MDA175	Triple neg		0.57	**−**	**−**	**−**	**−**				**−**	**−**	**−**	**−**
HMEC1585	Control		0.54	**−**	**−**	**−**	**+**				**−**	**−**	**−**	**+**
CALU3	Control		**12.59**	**−**	**−**	**+**	**+**				**−**	**−**	**+**	**+**
NC11	Control		1.75	**−**	**−**	**−**	**−**				**−**	**−**	**−**	**−**
DNA20	Control		2.00											

# Gene copy number determined using DNA20 (from normal lymphocytes) as a diploid control; bold values indicate amplification.

* mRNA expression quantified in comparison to the immortalized breast line HMEC1585; Calu3 was used a positive control for *ERBB2*, and MCF7 for *ESR1*.

### RNA and DNA isolation

Cells were grown to 70–80% confluence, then harvested for total RNA and genomic DNA. For HCC lines, RNA was prepared using the Qiagen RNeasy Midi Kit (Qiagen, Valencia, CA) and DNA by phenol/chloroform extraction. For all other lines, RNA was isolated using Trizol (Invitrogen, Carlsbad, CA) according to the manufacturer's protocol, and DNA using the Blood Cell Maxi Kit (Qiagen).

### *ERBB2* copy number assessment by quantitative PCR

*ERBB2* copy number was quantified by real-time quantitative PCR (Q-OCR), using the Chromo4 PCR System (Bio-Rad Laboratories, Hercules, CA). *GAST*, located at 17q21 (on the same chromosomal arm as *ERBB2*) was used as a reference control. PCR primer sequences for *ERBB2* and *GAST* are as follows (forward and reverse, respectively): *ERBB2*( 5′-TTGGGAGCCTGGCATTTCT-3′ and 5′-AGGTCATCGTGCCCACTCTT-3′); *GAST* (5′-GTAGGCATCCTTCCCCCATT-3′ and 5′-AGCCATGGTCCCTGCTTCTT-3′), with PCR product lengths of 59 and 70 base pairs, respectively. Primers were chosen by TaqMan Primer Express™ 1.5 (Applied Biosystem, Foster City, CA) and purchased from Invitrogen. PCR reactions were carried out in a final volume of 20 µl containing 20 ng genomic DNA, 300 nM each primer (for both *ERBB2* and *GAST*, in independent reactions) and 1× Power SYBR Green PCR Master Mix (Applied Biosystems, Foster City, CA). PCR conditions were as follows: one cycle at 95°C for 10 minutes, followed by 40 cycles each at 95°C for 15 seconds and 60°C for 1 minute. Samples were analyzed in triplicate. Each amplification reaction was checked for the absence of nonspecific PCR products by melting curve analysis. *ERBB2* copy number calculation was carried out using the comparative Ct method [8] after validating that the efficiencies of PCR reactions of both *ERBB2* and *GAST* were equal. Human Genomic DNA (DNA20) (EMD Biosciences, Darmstadt, Germany), a mixture of pooled human whole blood from 6–8 individual male and female donors, was run in every assay as a calibrator sample. *ERBB2* gene copy number in normal human genomic DNA was set as 2 and copy number more than 4 in cell lines was considered to be increased.

### mRNA levels of *ESR1, PGR, ERBB2* and *EGFR*

Transcript levels of *ESR1, PGR, ERBB2* and *EGFR* were analyzed as a part of RT2 Profiler Custom PCR Array (SuperArray Bioscience, Frederick, MD). After making cDNA from 1.0 µg total RNA using RT2 PCR Array First Strand Kit (SuperArray Bioscience), quantitative PCR was performed with the Chromo4 PCR System (Bio-Rad Laboratories) using RT2 Real-Time SYBR Green PCR Master Mix (SuperArray Bioscience) according to the manufacturer's protocol. We chose two different housekeeping genes, β-actin (*ACTB*) and glyceraldehyde-3-phosphate dehydrogenase (*GAPDH*) as internal controls, using the average of their Ct values. Primers were chosen by Taqman Primer Express™ 1.5 and purchased from Invitrogen, as follows: (forward and reverse, respectively): *ESR1* (5′-ATCTCGGTTCCGCATGATGAATCTGC-3′ and 5′-TGCTGGACAGAAATGTGTACACTCCAGA-3′); *PGR* (5′-CCTGTGGGAGCTGTAAGGTCTT-3′ and 5′-GCAGTCATTTCTTCCAGCACATA-3′), *ERBB2* (5′-TGACCTGCTGGAAAAGGGGGAGCG-3′ and 5′-TCCCTGGCCATGCGGGAGAATTCAG-3′); *EGFR* (5′-ATAGTCGCCCAAAGTTCCGTGAGT-3′ and 5′-ACCACGTCGTCCATGTCTTCTTCA-3′); *ACTB* (5′ GGCTGTGCTGTGGAAGCTAAG-3′ and 5′-ATGATGGAGTTGAAGGTAGTTTCGT-3′) [9]. We also analyzed the values of NC11 (normal lymphocyte) cell line for *ESR1, PGR, ERBB2* and *EGFR* mRNA expression, and the tumor cell values were reported relative to NC11. For data analysis, the comparative Ct method [8] was used.

### Western blot analysis and immunohistochemistry (IHC)

Preparation of total cell lysates and Western blotting were done as described previously [10]. Primary antibodies used were mouse monoclonal anti-ER-α (Cell Signaling, Beverly, MA), mouse monoclonal PR (6A1) (Cell Signaling), mouse monoclonal anti-HER2 (Cell Signaling), rabbit monoclonal anti-EGFR (Cell Signaling) and mouse monoclonal anti-actin (Sigma-Aldrich). Actin levels were used as a control for protein loading. Peroxidase-labeled anti-mouse or anti-rabbit antibodies (Amersham Pharmacia, Piscataway, NJ) were used as secondary antibody. IHC on breast cancer cell lines was described previously [11].

### *BRCA1* mutation analysis

DNA sequence analysis was performed on the entire *BRCA1* gene in available lymphocyte DNA matched to breast cancer cell lines. In the lymphocyte DNA matching HCC3153, a heterozygous duplication of 10 base pairs was detected at position 943 in exon 11 of *BRCA1* (943ins10). The region of BRCA1 exon 11 containing the 943ins10 mutation was amplified from genomic DNA in the tumor cell line (HCC3153) using standard PCR conditions. Sequence analysis revealed only the mutant sequence. Absence of the normal allele was also confirmed by single strand conformation analysis as well as gel electrophoresis of the amplified fragment on 5% acrylamide denaturing gels.

### Gene expression profiling

Gene expression profiling was performed on Human Exonic Evidence Based oligonucleotide (HEEBO) arrays obtained from the Stanford Functional Genomics Facility and containing 36,192 oligonucleotides representing 18,141 mapped human genes. 40 µg of sample RNA and 40 µg of “universal” reference RNA (derived from 11 different established human cell lines) were differentially labeled with Cy5 and Cy3, respectively, using an amino-allyl coupling protocol, then cohybridized onto the microarray in a high volume mixing hybridization at 65°C for 40 hrs. Details of the array processing and sample labeling/hybridization methods have been described [12]. Following hybridization, arrays were washed and scanned using a GenePix 4000B Axon scanner (Axon Instruments, Union City, CA). Fluorescence ratios were extracted using Spot Reader software (Niles Scientific, Portola Valley, CA) and uploaded to the Stanford Microarray Database [13] for storage, retrieval, and analysis. For two lines, HCC1806 and SUM44PE, expression profiling array hybridizations did not meet quality-control inspection and were excluded from analysis. The complete microarray expression data are available at the Stanford Microarray Database (SMD) (http://smd.stanford.edu) and at the Gene Expression Omnibus (GEO) (accession GSE15376); all microarray data reported in the manuscript are described in accordance with MIAME guidelines.

### Gene expression profiling analysis

Background-subtracted fluorescence log_2_ ratios were globally normalized for each array, and then mean-centered for each gene (i.e. reporting relative to the average log ratio across all samples). Unless otherwise specified, we included for subsequent analysis only well-measured genes defined as those with fluorescence intensities in the Cy5 or Cy3 channel at least 1.5-fold above background in at least 60% of samples. For unsupervised hierarchical clustering, we included only the 8,750 well-measured genes whose expression varied at least 3-fold from the mean in at least 5 samples (Table S1). Hierarchical clustering was performed and displayed using Cluster and TreeView software (http://rana.lbl.gov/EisenSoftware.htm). Enrichment for functionally related genes was tested across a collection of 1,687 curated gene sets (C2) using Gene Set Enrichment analysis (GSEA; Release 2.0) [14]. Cell lines were classified according to breast tumor subtype (luminal-A, luminal-B, ERBB2, basal-like and normal-like) using the nearest centroid method applied to the set of “intrinsic genes” (i.e. genes with small within-specimen compared to between-specimen expression variance), as done previously [15], here using Euclidean distance. To classify breast tumors (from the Sorlie *et al*. dataset [3]) according to cell line subtype (luminal, basal A, or basal B), we first built a classifier by combining the top 100 genes positively and negatively correlating with each of the three “one *vs*. others” cell line subtype distinctions, using Significance Analysis of Microarrays (SAM) [16]. The cell line subtype classifier, comprising 484 genes, was then applied to classify primary tumors using the nearest centroid method (with Euclidean distance). We also classified each cell line as being associated with a good or bad prognosis signature (70-gene prognostic signature [17]), the presence or absence of a wound healing signature (512-gene wound signature [18]), and the presence or absence of an hypoxia signature (123-gene hypoxia signature [19]). For each signature, we calculated the gene expression centroid of the two groups of breast tumors (as determined in the original publications), and then correlated each centroid with cell line expression of the respective signature genes. Membership was assigned to the group with the highest correlation (Pearson correlation).

### Array-based comparative genomic hybridization (aCGH)

Arrays for CGH were obtained from the Stanford Functional Genomics Facility. aCGH was performed using cDNA arrays containing 39,632 cDNAs, representing 22,279 mapped human genes (18,049 UniGene clusters [20], together with 4,230 additional mapped ESTs not assigned to UniGene IDs), according to previously published protocols [21], [22]. Briefly, 4 µg of genomic DNA from cell lines was random-primer labeled with Cy5 and co-hybridized onto a microarray along with 4 µg of Cy3 labeled normal leukocyte female reference DNA. Following overnight hybridization, the arrays were washed and scanned as above. The complete aCGH data are available at SMD and at GEO (accession GSE15376).

### aCGH analysis

Background-subtracted log_2_ fluorescence ratios were normalized for each array by mean centering. Well-measured genes used for subsequent analysis were those with fluorescence intensities in the Cy3 reference channel at least 1.4 fold above background. Map positions for arrayed cDNA clones were assigned using the NCBI genome assembly, accessed through the UCSC genome browser database (NCBI Build 36.1). For genes represented by multiple arrayed cDNAs, the average log_2_ ratio was used. The complete processed aCGH dataset is available as Table S2. DNA gains and losses were identified using the cghFLasso (R package for Fused Lasso) method [23], which controls the false discovery rate (FDR) by using normal-normal hybridization arrays to approximate the null distribution of the test statistics (see [23] for more details). A FDR<1% was used to call gains and losses. The fraction of the genome altered was determined by calculating the fraction of genes with fluorescence ratios ≥3 (for amplifications) or with significant non-zero fused lasso calls (for gains and losses). Some analyses (where indicated) were carried out on cytobands (boundaries defined by NCBI Build 36.1) rather than individual genes. For each cell line, cytobands exhibiting CNA were defined as those with at least two genes called by cghFLasso, and the magnitude of the CNA defined as the average log_2_ ratio of genes within the cytoband. We defined high-level DNA amplifications and multi-copy deletions as continuous regions identified by cghFLasso with at least 50% of genes having fluorescence ratios ≥3 or ≤0.25 respectively. These sites were also checked against known copy number variants (CNVs) reported in the Database of Genomic Variants (http://projects.tcag.ca/variation). Significant associations between cytobands and gene-expression subtypes were identified using SAM with a FDR<5%.

### Integrating genomic and transcriptional profiles

To integrate DNA copy number data (generated using cDNA microarrays) and gene-expression data (HEEBO oligonucleotide arrays), each gene expression measurement was first assigned a DNA copy number from either a probe interrogating the same named gene, or the average copy number of the nearest 5′ and 3′ probes (NCBI Build 36.1). Identification of genes with correlated copy number and expression was carried out using the DR-Correlate application of DR-Integrator (K. Salari, manuscript in preparation). Briefly, for each gene a modified Student's *t*-test was performed comparing gene expression levels in cell lines from the lowest and the highest deciles of all cell lines' copy number for the same gene; random permutations of sample labels were used to estimate a FDR.

## Results

### Transcriptional profiling identifies three breast cancer cell line subtypes

**Figure 1:**
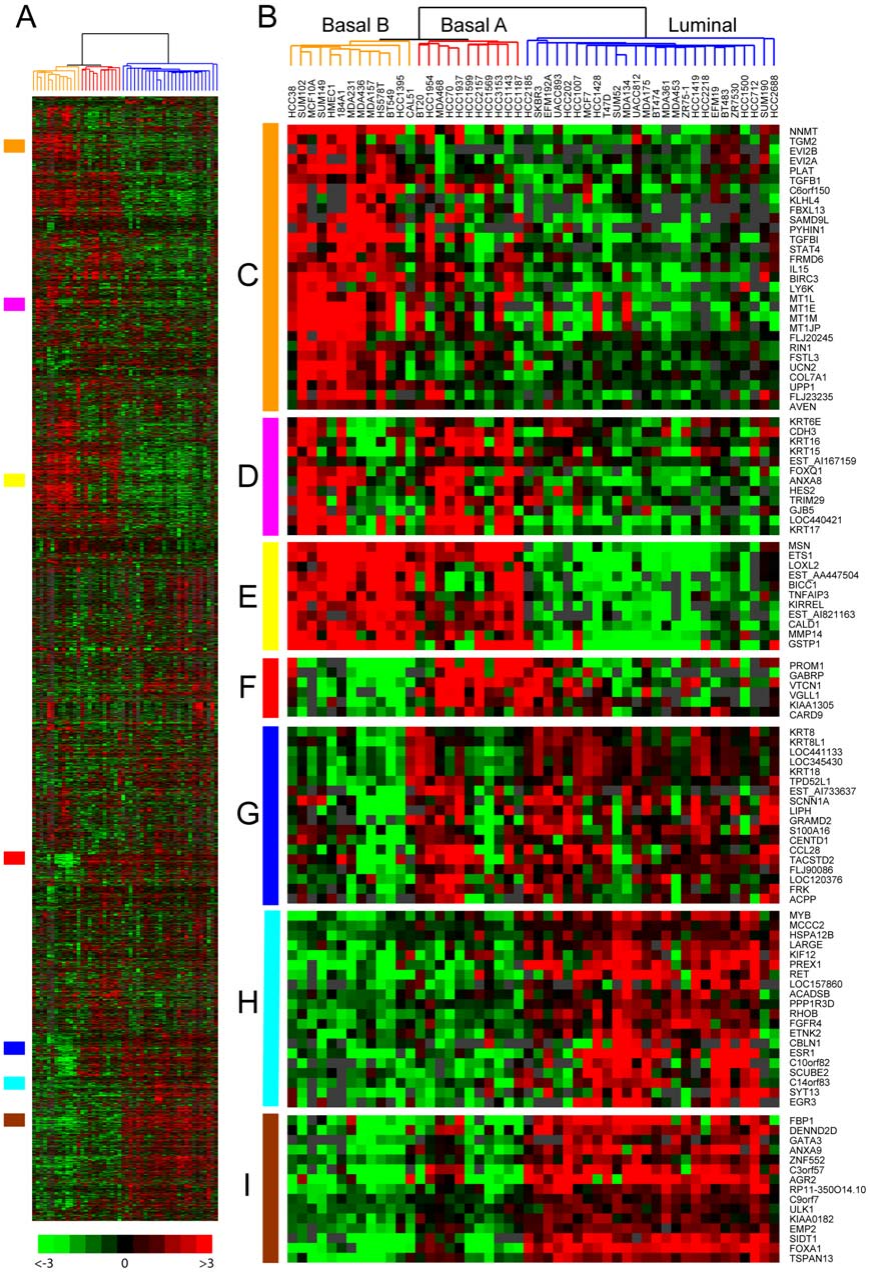
Clustering of expression profiles defines breast cancer cell line subtypes. (A) Thumbnail “heatmap” of two-way hierarchical clustering of 50 breast cancer cell lines (columns) and 8,750 variably expressed genes (rows) (data available as Table S1). Gene expression ratios are depicted by log_2_ pseudocolor scale shown; gray represents poorly measured data. (B) Enlarged view of the sample dendrogram. Clustering stratifies cell lines into two main groups, luminal (blue dendrogram branches) and basal, the latter further subdivided into two subgroups, basal A (red) and basal B (orange). (C–I) Selected gene expression patterns extracted from the cluster; corresponding locations in the thumbnail are indicated by the vertical colored bars. (C) Basal-B; (D) Basal cytokeratins; (E) Basal; (F) Basal-A; (G) Luminal cytokeratins; (H) ER-associated; (I) Luminal differentiation.

**Figure 2:**
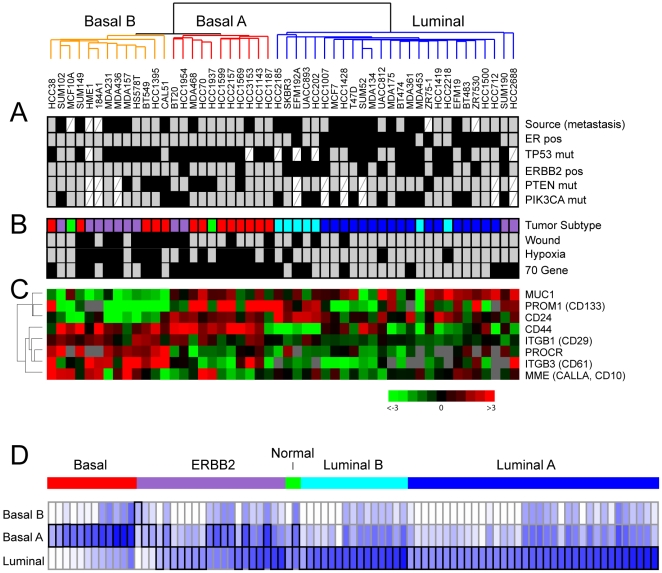
Subtype-specific expression and molecular characteristics. (A) Clinical, pathological and molecular characteristics of cell line expression subtypes. Black boxes indicate metastasis derivation, ER-positivity, *TP53* mutation, *ERBB2*/HER2 positivity, *PTEN* mutation, *PIK3CA* mutation. Mutation data compiled from the Sanger (http://www.sanger.ac.uk) and IARC (http://www-p53.iarc.fr) websites, and from refs. [94], [95]. White cross-hatched boxes indicate missing data. (B) Classification of cell lines by nearest resemblance to tumor gene-expression subtype: luminal A (dark blue), luminal B (light blue), ERBB2-associated (purple), basal-like (red) or normal-like (green); and by positivity (black boxes) for 70-gene, wound and hypoxia signature. (C) Expression levels of selected stem/progenitor cell relevant markers; log_2_ ratios are depicted by pseudocolor scale shown (gray represents poorly measured data). (D) Relation of tumor subtypes to cell line subtypes. Subtype of 86 tumors [3] is shown color-coded as above. Resemblance to each cell line subtype is depicted by Euclidian distance, indicated by blue intensity (representing shorter distances); best match is bracketed in black.

To catalog molecular variation in a collection of 52 widely-used breast cancer cell lines, we first profiled gene expression using whole genome oligonucleotide microarrays. Unsupervised hierarchical clustering of the 8,750 most variably expressed genes stratified cell lines into two main groups (see dendrogram, Fig. 1B). One group, designated “luminal” (blue dendrogram branches), contained all the ER-positive cell lines (Fig. 2A), and was characterized by the expression of ERα-regulated genes (e.g. *MYB*, *RET*, *EGR3*, *TFF1*; Fig. 1H, and not shown) [24]–[27], as well as genes associated with luminal epithelial differentiation (e.g. *GATA3* and *FOXA1*, Fig. 1I) [28].

The other group, designated “basal”, contained only ER-negative cell lines (Fig. 2A) and was characterized by the expression of basal epithelial gene markers including MSN, ETS1, CAV1 and EGFR (Fig. 1E, and not shown) [29]–[32]. Basal cell lines were further stratified into two subgroups, designated A and B (in line with Neve *et al*. [33], discussed further below). The basal-A subtype (red dendrogram branches) contained many of the “HCC” lines established at UT Southwestern, including two known *BRCA1* mutant lines (HCC1937, HCC3153) ([34], and this study). Basal-A lines were characterized by expression of PROM1 (aka CD133), a marker of various cancer stem cells [35], as well as other genes like *GABRP* and *VTCN1* (Fig. 1F and 2C). Some of the basal-A lines also shared expression of luminal epithelial markers like KRT8 and KRT18 (Fig. 1G).

The basal-B subtype (orange dendrogram branches) included non-tumorigenic lines (MCF10A, hTERT-HME1, 184A1) as well as several highly invasive lines exhibiting features of epithelial-mesenchymal transition (EMT) (MDA-MB231, MDA-MB436, MDA-MB157, Hs578t) [36]. Basal-B lines were characterized by markers associated with aggressive tumor features, including PLAT (plasminogen activator) [37] and TGFB1 [38] (Fig. 1C), as well as marker phenotypes associated with normal breast and breast cancer progenitor/stem cells (MUC^−^/CALLA^+^; CD44^+^/CD24^−/low^; and ITGB3(CD61)^+^) (Fig. 2C) [39]–[41]. In contrast to other basal lines, the subset of mesenchymal-like basal-B lines lacked expression of basal cytokeratin markers KRT5 and KRT17 (Fig. 1D, and not shown).

Subtype-specific differences in gene expression could also be identified by pathway analysis, using Gene Set Enrichment Analysis (GSEA) [14]. Included among the top signature associations (Table 3), the luminal cell line subtype was characterized by enriched expression of ER and good prognosis signatures, basal-A by ETS pathway and BRCA1 signatures, and basal-B by EMT and epidermal growth factor (EGF) signatures.

**Table 3:** GSEA of breast cancer cell line subtypes.

Subtype	Gene Set	Description	Source	FDR*
Luminal	BRCA_ER_POS	Correlated with ER+ in breast cancer	[17]	0.017
	BRCA_PROGNOSIS_POS	Correlated with good prognosis in breast cancer		0.094
Basal-A	ETSPATHWAY	ETS transcription factor pathway	BioCarta	0.063
	BRCA_BRCA1_POS	Correlated with BRCA1 (germline) in breast cancer	[17]	0.063
	IFN_ALL_UP	Upregulated with interferon-α,β,γ treatment	[96]	0.071
	IFNALPHA_HCC_UP	Upregulated with interferon-α treatment	[97]	0.076
	GLYCOGEN	Glycogen processing	Broad Institute	0.078
Basal-B	JECHLINGER_EMT_UP	Upregulated in EMT	[98]	0.040
	EGF_HDMEC_UP	Upregulated with EGF treatment	[99]	0.042
	DORSEY_DOXYCYCLINE_UP	Upregulated with GAB2 expression	[100]	0.047
	HTERT_DN	Downregulated with hTERT-immortalization	[101]	0.048
	HINATA_NFKB_UP	Upregulated by NF-κB	[102]	0.049

* Only top five significant gene sets shown.

In regard to molecular markers and gene mutations (Fig. 2A), the luminal subtype included all the ER-positive cancer lines (*P*<0.001, 2-tailed Fisher's exact test), and all but two of the ERBB2-positive lines (*P* = 0.002), half of which were also ER-positive. *PTEN* inactivating mutations and *PIK3CA* activating mutations, functioning on the same pathway, were mutually exclusive in all but one sample. Interestingly, *PTEN* mutations were more common in the combined basal-like cell lines (*P* = 0.020), while *PIK3CA* mutations were more frequent in luminal lines (*P* = 0.022). *TP53* mutations occurred more often in basal-like lines (*P* = 0.038).

### Relationship of breast cancer cell line and tumor subtypes

To determine the relation between breast cancer cell line subtypes (luminal, basal-A, basal-B) and breast tumor subtypes (luminal-A, luminal-B, ERBB2, basal-like, and normal-like), we first classified cell lines according to tumor subtype using a nearest centroid approach applied to the set of “intrinsic genes” used originally to define the tumor subtypes [2], [3] (see Methods) (Fig. 2B). By expression patterns, most of the luminal lines most closely resembled either luminal-A or luminal-B tumors. Most basal-A lines resembled basal-like tumors, and most basal-B lines resembled either basal-like or ERBB2 tumors (despite that none were ERBB2-positive).

We also carried out the reverse analysis, building a cell line subtype classifier to classify 86 breast tumors (from the original Stanford/Norway study defining the five tumor subtypes [3]) according to cell line subtype (see Methods) (Fig. 2D). Notably, all basal-like tumors most resembled basal-A cell lines. Luminal-A and -B tumors most resembled luminal cell lines, while ERBB2 subgroup tumors most resembled either luminal or basal-A cell lines. A similar analysis of breast tumors arising in carriers of *BRCA1* mutation, analyzed from a different dataset (The Netherlands Cancer Institute) [17], revealed highest resemblance in 17 of 18 cases to basal-A lines (not shown), while two *BRCA2* mutation associated cases most resembled luminal cell lines.

In addition to the above cluster-derived luminal/basal tumor subtypes, alternative breast tumor subtype classifiers have been proposed, including a 70-gene prognostic signature supervised on the metastatic/non-metastatic distinction [17], a “wound” signature trained on the serum response of cultured fibroblasts [18], and a hypoxia signature derived from the hypoxic response of cultured mammary and renal tubular epithelial cells [19]. Each of the three signatures predicts unfavorable clinical outcome. Interestingly, the basal-like lines (considered together) were those predominantly expressing the 70-gene (*P* = 0.001, Fisher's exact test) wound (*P* = 0.004), and hypoxia (*P*<0.001) signatures (Fig. 2B).

### Genomic profiles of breast cancer cell lines

To survey DNA copy number alterations in the panel of 52 breast cancer cell lines, we carried out CGH on cDNA microarrays with validated performance characteristics [21] and covering 22,000 genes with an average mapping resolution (inter-probe distance) of <70 Kb. Across the sample set, the most frequent CNAs (called by cghFLasso–see Methods) were gains on 1q, 3q, 5p, 7p, 8q, 11q, 17q, and 20q, and losses on 3p, 4, 8p, 9p, 11q, 13q, 18p, and Xq.

**Figure 3:**
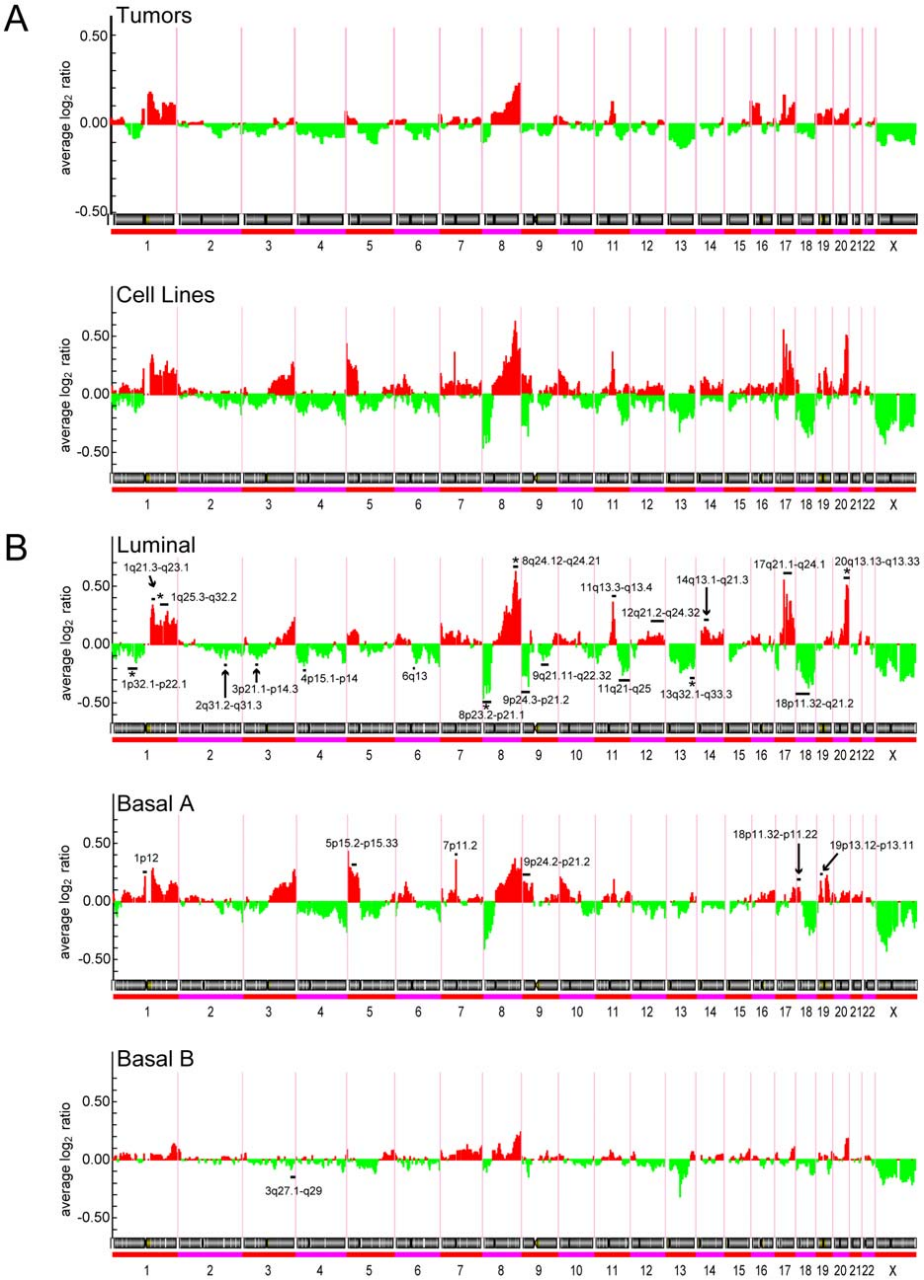
Genomic profiles define spectra of CNAs in cell line subtypes. (A) Spectra of gains (red) and losses (green) across the genome, plotted as average log_2_ ratio, for 89 breast tumors [4], above, compared to the set of 50 cell lines (profiled for both expression and CNAs), below. (B) Spectra of gains and losses for the cell line subtypes: luminal (above), basal A (middle) and basal B (below). Statistically significant subtype-specific CNAs, called by SAM (FDR<5%), are marked by a black bar. The subset of those loci that also characterize the corresponding primary breast tumor subtype is marked by an asterisk.

Overall, the spectrum of cytoband gains and losses was similar in the cell lines compared to primary tumors (Fig. 3A), though the frequency of those CNAs was generally higher with the cell lines. Cell line subtype-specific CNAs could be identified by SAM analysis (Fig. 3B). Luminal cell lines were characterized by more frequent gains on 1q, 8q, 11q, 12q, 14q, 17q and 20q, and losses on 8p, 9p, 11q, 13q, and 18p. Of these, gains on 1q, 8q, and 20q, and losses on 1p, 8p and 13q (asterisked in Fig. 3B) also characterize luminal-B breast tumors, while 17q gain characterizes ERBB2-associated tumors [4], [5]. Notably, simple patterns characteristic of luminal-A tumors (1q+, 16p+, 16q−) were not well-represented among the luminal cell lines. Basal-A and basal-B cell lines also exhibited characteristic gains/losses (Fig. 2B), but none also selectively characteristic of basal-like tumors.

**Figure 4:**
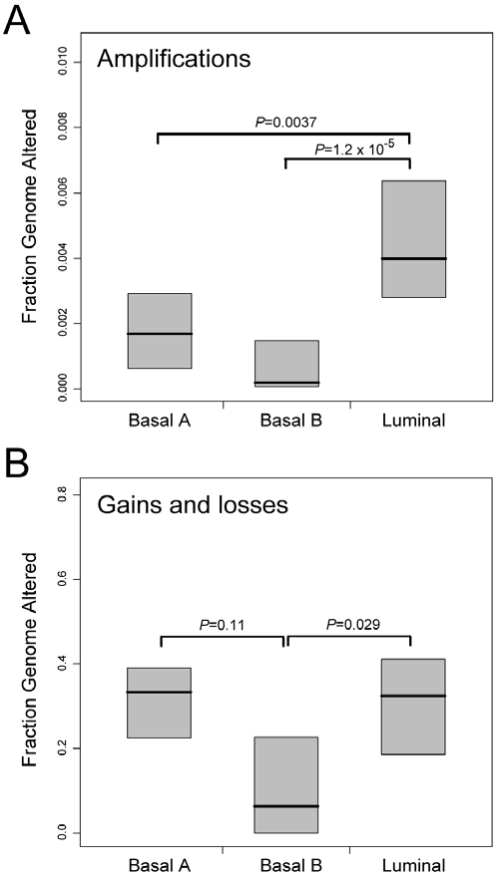
Cell line subtypes exhibit distinct genomic instabilities. Fraction of genome comprising (A) high-level DNA amplification; or (B) low-level gain/loss, stratified by cell line subtype (luminal, basal-A, basal-B). Box plots show 25^th^, 50^th^ and 75^th^ percentiles; *P*-values (Students *t*-test) for pairwise comparisons are shown.

Luminal cell lines displayed overall higher frequencies of high-level DNA amplification (i.e. fluorescence ratios ≥3, corresponding to at least 5-fold amplification [21]) (Fig. 4A), a characteristic shared with luminal-B tumors [4]. Luminal and basal-A lines both exhibited overall higher frequencies of gain/loss (a characteristic feature of basal-like tumors [4]), compared to basal-B lines (Fig. 4B).

### Integrated analysis for cancer gene discovery

The molecular profiles generated provide opportunities to identify breast cancer cell lines with an altered copy number and expression of known cancer genes, useful to model pathogenesis and therapy, and to discovery new breast cancer genes. For the latter, high-amplitude CNAs, i.e. high-level DNA amplifications and homozygous deletions, are particularly informative in pinpointing new cancer genes. Within the aCGH dataset we identified 80 loci of high-level amplification in 35 different cell lines, each spanning 49–49,014 Kb (median 1,115 Kb). We also identified 13 multi-copy (possibly homozygous) deletions (fluorescence ratios ≤0.25) in 8 cell lines spanning 132–7,825 Kb (median 1,477 Kb). The boundaries of amplicons/deletions did not correspond to known germline CNVs (reported in the Database of Genomic Variants), and, for the subset of recurrent alterations, finding distinct boundaries in different cell lines was more consistent with somatic alteration. Several regions of high-level amplification contained known oncogenes, like 8q24 (*MYC*), 11q13 (*CCND1*) and 17q12 (*ERBB2*). Other amplicons did not correspond to known oncogenes and presumably harbor novel breast cancer genes.

Gains and losses contribute to breast cancer by the increased and decreased expression of oncogenes and tumor suppressors, respectively. Using DR-Correlate (see Methods), we identified 3,511 genes (∼18% of all well-measured genes) whose altered expression correlated significantly (FDR<0.05) with altered gene copy number (Table S3). Of these, 487 resided within loci of high-amplitude CNA (Table 4). This subset included known breast cancer genes, like *EGFR* (7p11), *FGFR1* (8p12), *ERBB2* (17q12), *PPM1D* (17q23) and ZNF217 (20q13). This subset is likely also enriched for novel breast cancer genes, and as such represents a rich source for cancer gene discovery. Notably, among the larger group of amplified/overexpressed genes are several with known functions relevant to oncogenesis, like cell proliferation (e.g. *EIF3H*, *HEY1*, *MELK*, *GAB2*, CDC6, *GRB2*) [42]–[47], survival (e.g. *HIPK1*, *MCL1*, *MAPKAPK2*, *VCP*, *VDAC2*, *APIP*, *MAP3K3*) [48]–[54], migration/invasion (e.g. *MUC1*, *ADAM9*, *SH3PXD2A*, *CD44*, *PAK1*, *GIT1*, *PTPN1* ) [55]–[61], ER-signaling (e.g. *BCAS2*, *MUC1*, *NCOA3*, *TFAP2C* ) [62]–[65], and maintenance of genome integrity (e.g. *NBN*, *RAD21*, *FANCG*, *BUB3*, *RAD9A*, *TAOK1*, *RAD51C*, *RAE1*) [66]–[73]. Also represented are several “druggable” classes [74], like kinases (e.g. *HIPK1*, *MAPKAPK2*, *MELK*, *RPS6KB2*, *PAK1*, *TAOK1*, *PIP4K2B*, *RPS6KB1*, *TLK2*, *MAP3K3*), phosphatases (e.g. *PTPN1*), proteases (e.g. *ADAM9*), G protein-coupled receptors (e.g. *GPRC5C*) and ion channels (e.g. *VDAC2*).

**Table 4:** High-amplitude amplifications and deletions.

Cytoband	P-Border (nt)	Q-Border (nt)	Size (kB)	Cell Lines€	Significant DNA-RNA Correlations#	Other notable genes≠
**AMPLIFICATION**						
1p32.2	56946690	57156366	210	EFM192A		
1p22.1-1p21.3	93549298	97052934	3504	SUM44*	DR1, FNBP1L, ARHGAP29, ALG14	
1p13.3	107738670	109306637	1568	HCC2688	C1orf59, PRPF38B, STXBP3, GPSM2, CLCC1	VAV3
1p13.2	114220960	115183599	963	MCF7, UACC812	AP4B1, DCLRE1B, HIPK1, TRIM33, BCAS2, CSDE1, **NRAS**	
1q21.2	148738080	148885763	148	HCC1143	TARS2, **MCL1**, ENSA, GOLPH3L	
1q21.2-q21.3	149460307	150130540	670	HCC712, UACC812	PIP5K1A, PSMD4, ZNF687, PI4KB, PSMB4, POGZ, SNX27, MRPL9	
1q21.3	151000411	151885402	885	HCC712		
1q22	153424958	153999982	575	UACC812	MUC1, C1orf2, CLK2, HCN3, PKLR, C1orf104, RUSC1, ASH1L, YY1AP1	
1q23.3	159283361	159357995	75	SUM190	KLHDC9	
1q32.1	204736293	205144756	408	UACC812	MAPKAPK2	IKBKE
3p14.2-p14.1	61765808	64574645	2809	MCF7		
3q26.32	178223920	180535525	2312	HCC2185	TBL1XR1, ZNF639	PIK3CA
3q29	194971434	195513283	542	HCC1937		
3q29	196883266	196931777	49	HCC1937		
4q12	53304442	54084198	780	HCC1007	SCFD2, FIP1L1	
5p15.33	712977	2811691	2099	HCC1954	ZDHHC11, PDCD6, MRPL36, NDUFS6	TERT
6p12.1	55358212	57236103	1878	HCC1007	KIAA11586, ZNF451, BAG2	
6q16.3-q21	104858272	109112665	4254	HCC2185	HACE1, ATG5, C6orf203, PDSS2, SEC63, OSTM1, SNX3, FOXO3A	
6q21-q22.31	111961945	123089199	11127	HCC2185	C6orf225, HDAC2, DSE, GOPC, NUS1, ASF1A, HSF2, SERINC1	
7p15.2	26557965	27107611	550	HCC1007		
7p11.2	54595526	55931398	1336	BT20, MDA468	**EGFR**	
7q21.13-q21.2	90779687	91868629	1089	SUM52	MTERF, AKAP9, CYP51A1, KRIT1, ANKIB1	
7q21.3	95239813	96489919	1250	SUM52	SLC25A13, SHFM1	
7q22.1	100294293	100421513	127	SUM52	SLC12A9	
8p21.3	21593811	21966432	373	MDA134	XPO7	
8p12-p11.21	32328805	41907423	9579	BT483, HCC1500, HCC1599, MDA134, SUM44*, SUM52	FUT10, C8orf41, MAK16, ZNF703, ERLIN2, PROSC, BRF2, RAB11FIP1, EIF4EBP1, ASH2L, LSM1, BAG4, DDHD2, WHSC1L1, LETM2, **FGFR1**, TACC1, PLEKHA2, TM2D2, ADAM9, GOLGA7, AGPAT6	IKBKB
8q12.2-q12.3	61817956	62960675	1143	SUM190	CHD7	
8q13.3	71707355	72999610	1292	SKBR3		
8q21.11-q21.13	79781799	85260376	5479	EFM192A, HCC1419, HCC1599, SKBR3	HEY1, TPD52, ZBTB10	
8q21.3-q22.1	89113344	95233478	6120	EFM192A, HCC1419, SKBR3	OSGIN2, NBN, DECR1, OTUD6B, RBM12B, TMEM67	
8q22.2-q22.3	100879473	101995283	1116	HCC1419, HCC2185	COX6C, POLR2K	
8q22.3	104311423	104550566	239	HCC1419	FZD6	
8q23.1-q24.21	108267427	131134620	22867	EFM192A, HCC1419, HCC1599, HCC2185, SKBR3, ZR75-30	EIF3E, TRPS1, EIF3H, C8orf53, RAD21, TAF2, DSCC1, MRPL13, MTBP, DERL1, WDR67, C8orf76, ZHX1, ATAD2, C8orf32, FAM91A1, TMEM65, TRMT12, RNF139, TATDN1, NDUFB9, SQLE, KIAA0196, NSMCE2, FAM84B	MYC
8q24.22	133917771	134337653	420	ZR75-30	PHF20L1	
8q24.3	141658961	143348731	1690	HCC1419, MDA436, ZR75-30	GPR20, FLJ43860	
8q24.3	144310706	144753628	443	MDA436, ZR75-30	ZFP41, GLI4, ZNF696, C8orf51, RHPN1, MAFA	
8q24.3	145137850	146252219	1114	BT483, HCC1419, MDA436, ZR75-30	GRINA, OPLAH, SHARPIN, KIAA1833, FBXL6, CPSF1, VPS28, KIFC2, ZNF252	
9p13.3-p13.2	33876876	38058023	4181	HCC2185	UBE2R2, UBAP2, WDR40A, KIF24, KIAA1161, DCTN3, GALT, IL11RA, VCP, FANCG, PIGO, STOML2, RUSC2, TESK1, CD72, C9orf100, TLN1, CREB3, RGP1, HINT2, CLTA, RNF38, MELK, ZCCHC7, GRHPR, ZBTB5, POLR1E, FBXO10, RG9MTD3, WDR32, MCART1	
9q33.3	128307884	129195638	888	SUM44*	RALGPS1	
10q21.1-q21.2	72507196	73797267	1290	HCC2157	DNAJB12	
10q22.2-q22.3	76461776	82106491	5645	EFM19, HCC2157	SAMD8, VDAC2, DLG5, POLR3A, RPS24, LOC283050, ZMIZ1, PPIF, SFTPA1, FAM22E, C10orf57, ANXA11	
10q24.33-q25.1	105307581	106054698	747	EFM19	SH3PXD2A	
10q26.13	124598599	124962466	364	SUM52	IKZF5, BUB3	
11p13	33062705	35600197	2537	HCC1806*	HIPK3, FBXO3, CAPRIN1, NAT10, ABTB2, CAT, APIP, PDHX, CD44	
11q13.2	66874536	67198753	324	MDA134, ZR75-1	RAD9A, RPS6KB2, CORO1B, TMEM134	
11q13.3-q13.4	68427956	70812048	2384	HCC1143, HCC1500, HCC1954, MDA134, MDA175, MDA361, SUM44*, SUM190,	IGHMBP2, FADD, PPFIA1, CTTN, SHANK2	CCND1
11q13.4	73316198	73649077	333	BT474, MDA134, SUM190	UCP2, C2CD3, PPME1	
11q13.4-q14.1	74648813	77963474	3315	MDA134, SUM44*, SUM52, SUM190	ARRB1, PRKRIR, **EMSY**, PHCA, PAK1, AQP11, CLNS1A, C11orf67, INTS4, NDUFC2, ALG8, GAB2, NARS2	
12p12.3	18727378	19246201	519	HCC1500		
12q21.31-q21.33	88265969	88443930	178	SUM52	WDR51B, GALNT4	
13q22.2-q31.1	74756931	78096263	3339	UACC812	UCHL3	
13q31.3-q32.1	90798074	93942902	3145	UACC812		
16q12.2	51800892	53524601	1724	EFM19, SUM44*	CHD9, FTO	
17p12	12611513	13636592	1025	EFM192A	ELAC2	
17q11.2	23686912	24013273	326	ZR75-30	POLDIP2, TREM199, SLC46A1, PIGS, SPAG5, FLJ25006, KIAA0100, SDF2	
17q11.2	24894649	25818484	924	HCC202	TAOK1, LOC116236, GIT1, ANKRD13B, CPD	
17q11.2	27727543	28293356	566	SUM190	ZNF207	
17q12	31206068	31649844	444	MDA361	FLJ12120	
17q12-q21.2	32627885	36209712	3582	BT474, EFM192A, HCC202, HCC1419, HCC1569, HCC1954, HCC2218, MDA361, SKBR3, SUM190, UACC812, UACC893, ZR75-30	ACACA, TADA2L, DDX52, SOCS7, MLLT6, CISD3, PCGF2, PSMB3, PIP4K2B, CCDC49, RPL23, LASP1, CACNB1, FAM153C, RPL19, LOC90110, FBXL20, MED1, PPP1R1B, STARD3, TCAP, PERLD1, **ERBB2**, C17orf37, GRB7, IKZF3, GSDML, ORMDL3, PSMD3, MED24, MSL-1, CASC3, CDC6, RARA, SMARCE1	
17q21.31	38419019	38738864	320	SUM190	RND2	
17q21.32-q25.1	43329972	50826668	7497	BT474, EFM192A, HCC202, HCC712, HCC1419, HCC2218, ZR75-30	SP2, PNPO, CDK5RAP3, SNX11, HOXB13, CALCOCO2, ATP5G1, UBE2Z, SNF8, ZNF652, PHB, SPOP, SLC35B1, FAM117A, MYST2, PDK2, XYLT2, MRPL27, LRRC59, EME1, ACSF2, RSAD1, EPN3, SPATA20, ABCC3, ANKRD40, CROP, TOB1, NME1, TOM1L1, COX11, STXBP4	
17q23.2-q24.2	53282667	63106134	9823	BT474, HCC712, HCC2218, MCF7, MDA361, ZR75-30	SFRS1, DYNLL2, MKS1, SUPT4H1, MTMR4, RAD51C, TRIM37, FAM33A, C17orf71, YPEL2, DHX40, CLTC, PTRH2, TMEM49, TUBD1, **RPS6KB1**, RNFT1, HEATR6, USP32, APPBP2, **PPM1D**, BRIP1, INTS2, MED13, METTL2A, TLK2, TANC2, CYB561, WDR68, CCDC44, MAP3K3, LYK5, CCDC47, DDX42, PSMC5, SMARCD2, DDX5, CCDC45, SMURF2, GNA13, HELZ	
17q25.1	69755691	71418122	1662	HCC2218, MDA361, MDA453, UACC893	GPRC5C, SLC9A3R1, NAT9, TMEM104, FDXR, C17orf28, CDR2L, ICT1, KCTD2, SUMO2, NUP85, GGA3, MRPS7, MIF4GD, SLC25A19, GRB2, CASKIN2, TSEN54, MYO15B, SAP30BP, H3F3B, UNK, WBP2	
18q21.32-q21.33	55178911	57628085	2449	HCC1500		
19p13.2	14932742	15602448	670	HCC1143	ILVBL, BRD4, AKAP8L	
19q12-q13.11	33966349	38052482	4086	HCC1569, HCC1599	UQCRFS1, POP4, PLEKHF1, C19orf2, DPY19L3, ANKRD27	
19q13.11	39866832	40146793	280	HCC1599		
19q13.42	60551045	60898029	347	EFM19	FIZ1, ZNF784, CCDC106	
19q13.43	63208125	63774724	567	HCC1806*	ZNF329, ZNF274, ZNF8, ZSCAN22, ZNF324, TRIM28, CHMP2A, UBE2M	
20p12.2	10224083	10433564	209	HCC2185	MKKS	
20q11.22	32363269	33563203	1200	BT474	DYNLRB1, NCOA6, UQCC	
20q13.12	42493067	43286511	793	BT474, SUM52	SERINC3	
20q13.12-q13.13	45234836	48636574	3402	BT474, HCC1419, MCF7	NCOA3, PREX1, ARFGEF2, STAU1, DDX27, ZNFX1, SLC9A8, SPATA2, PTPN1	
20q13.13-q13.32	49139330	57334442	8195	BT474, HCC1419, MCF7, SKBR3	ZFP64, **ZNF217**, BCAS1, PFDN4, C20orf108, CSTF1, C20orf43, TFAP2C, BMP7, RAE1, RBM38, RAB22A, VAPB, STX16, NPEPL1, GNAS, TH1L, ATP5E, SLMO2	AURKA
20q13.33	61801252	62370522	569	HCC1419	PRR17, OPRL1	
22q11.21	18256420	19686015	1430	SUM190	COMT, HTF9C, PI4KA	
22q12.1	24895479	25885840	990	HCC202	HPS4	
Xp11.23-p11.22	48635684	51225253	2590	HCC712		
Xp11.22	52255712	54236019	1980	HCC202	TMEM29, PHF8	
Xq28	148368959	149592006	1223	HCC202		
**DELETION**						
6q16.3-q21	102493055	105832848	3340	HCC1395	**HACE1**	
7q11.23-q21.11	77246720	77484743	238	HCC1806*	TMEM60, PHTF2	
8p23.3	604200	2080787	1477	HCC2688	ERICH1	
9p24.3-p24.2	958704	3213008	2254	HCC2185	VLDLR, KIAA0020	
9p21.2-p21.1	26894518	29207861	2313	BT474, EFM19	PLAA, IFT74	CDKN2A
13q14.3-13q21.2	52175620	60001053	7825	HCC1395		
15q24.3	74984799	75116728	132	HCC1806*	RCN2	
17p12	11405197	11987872	583	EFM19	MAP2K4	
17q21.31	38252285	38419019	167	HCC1806*		BRCA1
18q11.2-q12.1	22256956	23913060	1656	HCC2185		
21q21.1	18342236	21590772	3249	ZR75-30		
Xp11.3	46208136	46345060	137	HCC2157		
Xq25	122657657	123338533	681	HCC1806*		

€ For aberrations spanning multiple lines, inclusive interval indicated.

* DNA but not RNA profiled.

# Only named genes listed, ordered by genome position; bold text indicates select known cancer genes.

≠ Within or immediately flanking interval.

## Discussion

Using whole-genome DNA microarrays, we collected transcriptional and genomic profiles across a set of 52 widely used breast cancer cell lines, with the primary goals to establish their suitability in modeling known breast tumor heterogeneity, and to create a resource for cancer gene discovery. Cluster analysis of transcriptional profiles defined three cell line subtypes, one luminal and two basal (A and B), consistent with other recent studies of breast cancer cell lines [31], [33], [75]. The luminal subtype included all ER-positive cell lines, and associated gene expression patterns reflected both ER and luminal differentiation pathways, the latter including GATA3 and FOXA1, key transcriptional mediators of luminal differentiation [28], [76]. The basal-like cell lines were ER-negative and exhibited more frequent mutations of *TP53* and *PTEN*, consistent with findings in basal-like tumors [3], [77]. The basal-A subtype exhibited enriched expression of ETS pathway genes, a pathway linked to diverse tumor phenotypes including invasion and metastasis [78]. The basal-B subtype, which included the three non-tumorigenic lines (consistent with prior studies [75]), as well as five highly invasive/metastatic lines with features of EMT, exhibited enriched expression of EMT and EGF regulated genes, the latter pathway also previously linked to basal-like tumors [79].

Recently, Neve *et al*. [33] profiled 51 breast cancer cell lines (though using a lower-resolution (∼1 Mb) CGH platform), 38 of which (∼3/4^th^) overlapped with the 52 we profiled. All the overlapping lines except for one clustered into the same corresponding gene-expression subtype in both their and our study. The exception was HCC1500, which we classified as luminal while Neve *et al*. labeled it as basal B. The discrepancy may reflect a cell line identification error. We note that ATCC describes the line as ER-positive, more consistent with a luminal classification.

Our comparisons of expression profiles between breast cancer cell line subtypes and breast tumor subtypes provided valuable information relevant to the suitability of cell lines in modeling known breast tumor heterogeneity. Luminal-A/B tumors best matched luminal cell lines. Notably, basal-like tumors most corresponded to basal-A cell lines. Consistent with this finding, two breast cancer cell lines from *BRCA1* mutation carriers also clustered in basal-A (and basal-A lines exhibited enrichment of a BRCA1 signature), where it has been established that *BRCA1*-associated tumors share many features with sporadic basal-like tumors [80]. Interestingly, ERBB2-associated tumors matched both luminal and basal-A lines. While ERBB2 represents a distinct expression tumor subtype in multiple independent cohorts [3], [15], [81], it is noteworthy that most ERBB2 (HER2+) cell lines clustered in the luminal subtype. The basis for the discrepant ERBB2 grouping in cell lines and tumors is unclear but warrants further investigation.

It has been suggested that the origin of the luminal *vs*. basal breast cancer distinction reflects the transformation of different breast epithelial progenitor cell compartments [82], [83]. Breast epithelial stem/progenitor cells support mammary gland development during puberty and subsequent growth and remodeling during pregnancy [84]. A prevailing view is that breast epithelial stem cells give rise to bipotent basal/luminal progenitors, which then give rise to basal and luminal restricted progenitors, and from there to differentiated basal/myoepithelial and luminal epithelial cells [84], [85]. Bipotent human breast epithelial stem/progenitors have been characterized with the cell surface phenotype MUC^−/low^/CALLA^low/+^ [39]. Separately, breast cancer stem cells, identified prospectively as tumor initiating cells when transplanted into immunodeficient mice, have been characterized by the surface expression phenotype CD44^+^/CD24^−/low^ [40], also a presumed phenotype of normal breast epithelial stem or early progenitor cells [84].

Our transcriptional profiles of breast cancer cell lines are consistent with an origin in (or at least a likeness of the bulk cell population to) the various stem/progenitor cell compartments. Basal-B lines predominantly express CD44^+^/CD24^−/low^ and MUC^−^/CALLA^+^ phenotypes characteristic of stem or bipotent progenitor cells, as well as ITGB3 (CD61), also recently characterized as a cancer stem cell marker in MMTV-wnt-1 induced murine breast cancer [41]. In contrast, basal-A lines appear mainly CD44^+^/CD24^+^, but express PROM1 (aka CD133), a marker of luminal progenitors in mice [86] also more recently characterized as a stem cell marker in BRCA1-associated breast cancer [87], while luminal lines express markers of luminal lineage restriction like GATA3 and FOXA1 [28]. Conspicuously absent from our analysis is a breast tumor subtype corresponding to the stem-cell like (and sometimes mesenchymal-like) basal-B lines. Whether basal-B lines reflect an uncommon tumor subtype not yet characterized, or else a stem/progenitor subpopulation of tumor cells enriched in culture, or even an artifact of cell culture, remains to be determined. Regardless, breast cancer cell lines are likely to prove useful for discovering new stem cell markers, and for studying stem/progenitor cell biology.

Our genomic profiles of breast cancer cell lines indicate that overall the spectra of CNAs is reflective of breast tumors, consistent with prior findings from loss of heterozygosity (LOH) analysis [11]. Overall, however, cell lines exhibited higher frequencies and greater complexities of CNAs, and seemingly more than might be explained by a higher sensitivity of detecting CNAs in stromal-free tumor cell populations. Notably absent among the luminal subtype were the “simple” karyotypes characteristic of luminal-A tumors (i.e. 1q+, 16p+/16q−). By genomic profiles, luminal cell lines shared features characteristic of luminal-B tumors, including certain subtype-specific CNAs and overall higher levels of DNA amplification. Likewise, basal-A cell lines and basal-like tumors shared the feature of high levels of chromosome segment gain/loss. However, overall only a subset of subtype-specific CNAs was preserved. Therefore, at the genomic level it is uncertain how well cell line subtypes faithfully represent tumor subtype counterparts.

Taken together, the transcriptional and genomic profiles support the conclusion that luminal and basal-A cell lines are the most appropriate cell line models of luminal-B and basal-like tumors, respectively. Further, the basal lines are likely useful models for biological studies of the 70-gene, wound and hypoxia signatures. Despite incongruent expression results, luminal lines with amplification/overexpression of ERBB2 are likely appropriate models of ERBB2-associated tumors. Our findings indicate that new cell lines are needed to more faithfully model luminal-A tumors. Currently available cell lines likely reflect certain biases in the specimen source of cell line, and/or in the culturing methods, as suggested by the predominance of HCC lines (from UT Southwestern) among the basal-A group. Different culturing methods (e.g. ref. [88]) might support the establishment of cell lines from luminal-A tumors.

Our genomic profiles also identified numerous high-level DNA amplifications and multi-copy deletions, pinpointing known and novel cancer genes. Further, by integrating the genomic and transcriptional datasets, we could define a set of candidate cancer genes residing at these loci and exhibiting both altered copy number and expression. The larger set of amplified/overexpressed genes included several known breast cancer oncogenes, as well as many plausible candidates including genes with known functions relevant to carcinogenesis, like cell proliferation, survival and motility/invasion, and genome integrity (e.g. DNA damage response). Though genes maintaining genome integrity are more typically considered candidate tumor suppressors, the overexpression of such genes has been linked to genome instability [67], [89]. The set of amplified/overexpressed genes also included many druggable targets [74], most notably several kinases. Importantly, the same cell lines used for discovery can also be used to functionally examine cancer gene candidates, for example using RNA interference to knockdown the expression of amplified oncogene candidates, and then assaying loss of tumorigenic phenotypes in cultured cells or *in vivo* (e.g. refs.[90], [91]). Indeed, high-throughput RNA interference approaches [92], [93] might be used to evaluate many or all of the candidate cancer genes simultaneously.

In summary, transcriptional and genomic profiling of 52 commonly used breast cancer cell lines identifies cell line subtypes, and defines the cell line subtypes that most faithfully capture the known heterogeneity of breast tumors. Specifically, luminal and basal-A lines appear to best model the features of luminal-B and basal-like tumors, while basal-B lines might inform stem cell biology. In addition, our integrated analysis of genomic and transcriptional profiles pinpoints loci and genes with altered copy number and expression, providing a rich source for discovery and future characterization of new breast cancer genes.

## Supporting Information

Table S1 8,750 variably expressed genes (log2 ratios)
(1.11 MB ZIP)

Table S2 Processed aCGH data (log2 ratios)
(2.82 MB ZIP)

Table S3 Genes with significantly correlated copy number and expression
(0.13 MB TXT)
